# Effect of Mono- and Poly-CH/P Exchange(s) on the Aromaticity of the Tropylium Ion

**DOI:** 10.3390/molecules21081099

**Published:** 2016-08-20

**Authors:** Ankita Puri, Raakhi Gupta

**Affiliations:** Department of Chemistry, The IIS University, Jaipur 302020, India; ankita.puri@iisuniv.ac.in

**Keywords:** phosphatropylium ions, aromaticity, NICS, magnetic susceptibility exaltation, HOMO − LUMO gap

## Abstract

In view of the fact that the phosphorus atom in its low co-ordination state (coordination numbers 1 and 2) has been termed as the carbon copy, there have been attempts to investigate, theoretically as well as experimentally, the effect of the exchange(s) of CH- moiety with phosphorus atom(s) (CH/P) on the structural and other aspects of the classical carbocyclic and heterocyclic systems. Tropylium ion is a well-known non-benzenoid aromatic system and has been studied extensively for its aromatic character. We have now investigated the effect of mono- and poly-CH/P exchange(s) on the aromaticity of the tropylium ion. For this purpose, the parameters based on the geometry and magnetic properties, namely bond equalization, aromatic stabilization energies (ASE), Nucleus-Independent Chemical Shift (NICS) values, (NICS(0), NICS(1), NICS(1)_zz_), proton nucleus magnetic resonance (^1^H-NMR) chemical shifts, magnetic susceptibility exaltation and magnetic anisotropic values of mono-, di-, tri- and tetra-phosphatropylium ions have been determined at the Density Functional Theory (DFT) (B3LYP/6-31+G(d)) level. Geometry optimization reveals bond length equalization. ASEs range from −46.3 kcal/mol to −6.2 kcal/mol in mono- and diphospha-analogues which are planar. However, the ions having three and four phosphorus atoms lose planarity and their ASE values approach the values typical for non-aromatic structures. Of the three NICS values, the NICS(1)_zz_ is consistently negative showing aromatic character of all the systems studied. It is also supported by the magnetic susceptibility exaltations and magnetic anisotropic values. Furthermore, ^1^H-NMR chemical shifts also fall in the aromatic region. The conclusion that mono-, di-, tri- and tetra-phosphatropylium ions are aromatic in nature has been further corroborated by determining the energy gap between the Highest Occupied Molecular Orbital (HOMO) and Lowest Unoccupied Molecular Orbital (LUMO) (HOMO − LUMO gap), which falls in the range, ca. 3 × 10^−19^–9 × 10^−19^ J. The systems having more than four phosphorus atoms are not able to sustain their monocyclic structure.

## 1. Introduction

The concept of aromaticity is of fundamental importance for explaining the structure, stability and reactivity of many molecules, due to which it continues to attract attention of many eminent theoretical chemists and forms the basis of many interesting monographs [1,2,3], research papers [4,5,6,7,8,9] and thematic issues [10,11,12]. Hückel’s predictions based on the molecular orbital theory that conjugated monocyclic planar systems containing (4n + 2) π electrons should be aromatic, i.e., conjugatively stabilized [13], was modified by Platt [14], who broadened the scope of the postulation by including neutral as well as charged polycyclic systems in this category. The term “antiaromatic” was used in substitution of “pseudoaromatic” after 1965 by Breslow for those compounds that exhibited significant destabilization characteristics and possessed (4n) π conjugated electrons [15]. The possibility of verifying theoretical predictions with experimental isolations is limited to aromatic compounds only. Post-Hückel, there have been many attempts to propose newer and more reliable indicators to determine aromaticity/antiaromaticity of a particular system or a class of compounds. Schleyer et al. [16] endeavoured to divide various theoretical parameters into four categories, each based on a particular property:

Structure—tendency towards bond length equalization and planarity (if applicable).
Energy—enhanced stability.
Reactivity—lowered reactivity, electrophilic aromatic substitution (if applicable).
Magnetic properties—proton nucleus magnetic resonance (^1^H-NMR) chemical shift, magnetic susceptibility exaltation and anisotropies, nucleus-independent chemical shift (NICS), ring current plots.

In a recent review [17], geometry-based various aromaticity indices have been discussed elegantly. Bond length equalization has been perceived as one of the important manifestations of aromaticity, but it cannot be used as the sole criterion to characterize it unambiguously, as there are examples like borazine, which has equalized bond length but is not aromatic. The quantitative indices have been developed by Francois and Julg [18] and Bird [19], but extension of these indicators to the heteroaromatic compounds is not straightforward.

The aromatic stabilization energies (ASEs) and the enhanced resonance energies (REs) have been recognized to be the keystone of aromaticity since long ago. However, these energy estimates vary significantly, depending strongly on the equations used and on the choice of the reference molecules. As such, the other effects like hyperconjugative, etc. are also not taken into consideration or are neglected while calculating the ASEs and REs, and, therefore, in some specific cases, large discrepancy is noted. Schleyer et al. [20] have reported improved results by calculating isomerization stabilization energies (ISE) instead of ASEs.

The most reliable and commonly used indicators for validating aromaticity of different systems including the transition states are the magnetic criteria. The exalted diamagnetic susceptibility has been used to quantify aromaticity ever since it was proposed by Pascal in 1910 [21]. ^1^H-NMR chemical shifts are also used frequently for characterizing aromatic compounds [22]. The effect of the diatropic ring current induced in an aromatic molecule by an external magnetic field is much stronger inside the ring in comparison to outside. Schleyer et al. in 1996 proposed another indicator of aromaticity, namely NICS based on the magnetic shielding at the center of the aromatic ring caused by the induced diatropic current [23]. Subsequently, it was refined and magnetic shielding 1Å above the center of the aromatic ring (NICS(1)), the zz component of NICS(1) (NICS(1)_zz_), and dissected NICS were considered to be better indicators [16].

An impressive analogy between P=C and C=C double bonds in the molecules having several PC double bonds was emphasized for the first time by Appel [24] while describing Cope-rearrangement of several diphospha- and tetraphospa-hexadienes. Thereafter, similar conjugative ability of the P=C and C=C double bonds was reiterated by analyzing π-ionization energies and ring-fragmentation/isodesmic energies of a series of heterocyclic conjugated systems containing σ^2^,λ^3^-P atoms [25,26,27,28]. The analogy turned out to be so prolific that besides a few research papers [29,30,31,32,33] on this theme, a monograph with the title, “Phosphorus: The Carbon Copy” [34] has been published. The effect of the exchange of the CH moiety with phosphorus atom (CH/P exchange) on the aromaticity of the carbocyclic rings has been discussed in two recent reviews [35,36], which include detailed experimental and theoretical discussion of the three- to six-membered rings having one or more phosphorus atoms. It was concluded that due to the small angle at the P atom (ca. 95 degrees), aromaticity is sustained on CH/P exchange in the small rings, but in large rings, it results in reduced aromatic character and planarity is disturbed.

On the basis of the recent studies carried out by our research group [37,38], it was demonstrated that mono- and poly-phospha analogues of carbocyclic cations and anions, namely, cyclopropenium, cyclopropenide, cyclopentadienium and cyclopentadienide, display aromatic or antiaromatic character comparable with that of the respective carbocyclic ion. However, the results are not uniform in the case of antiaromatic systems.

The aromatic character of the tropylium cation and its monophospha- and other hetero-analogues has been probed earlier at the hybrid functional level using various structural and magnetic descriptors [39,40,41]. The tropylium cation was found to have a planar structure with completely delocalized 6π-electrons. The CH/P exchange in the tropylium cation was accompanied by lowering of the aromaticity only marginally.

As no attempt has been made so far to study the effect of more than one CH/P exchanges on the aromaticity of the tropylium cation, the present study concerns the investigation of the effect of mono- and poly-CH/P exchange(s) and to compare the results obtained on the basis of geometry, energetic and magnetic criteria. For this purpose, we computed the optimized geometries and calculated ASEs, ^1^H-NMR chemical shifts, NICS(0), NICS(1), NICS(1)_zz_, magnetic susceptibility exaltation and magnetic susceptibility anisotropy values and the energy difference of the highest occupied molecular orbital and lowest unoccupied molecular orbital (HOMO − LUMO energy gap) for the tropylium ion and its mono-, di-, tri- and tetra-phospha analogues at the Density Functional Theory (DFT) (B3LYP/6-31+G(d)) level. As discussed later, phosphatropylium ions having five or more phosphorus atoms do not sustain their monocyclic structures. These results are presented here. The corresponding anions will be discussed in a separate communication.

## 2. Results and Discussion

**Scheme 1:**
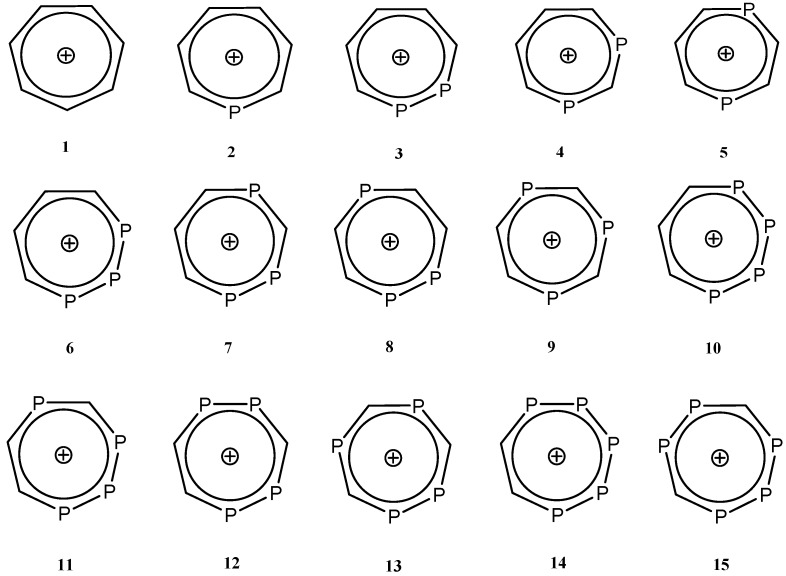
Tropylium ion and its mono- and poly-phospha analogues.

The following model cations (Scheme 1) were computed at the B3LYP/6-31+G(d) level to study the effect of the CH/P exchange on the aromaticity of the tropylium ion.

### 2.1. Optimized Geometries

The Julg concept [18] is based on bond length equalization in the aromatic systems. However, it could not be extended to the heteroaromatic systems. To overcome this difficulty, the Bird index [19] was developed, but that too involved cumbersome calculations involving several aromaticity indices as constants. In the present study, our discussion is limited to the equalization or localization of the bonds without resorting to the calculation of the Julg or Bird indices.

**Figure 1:**
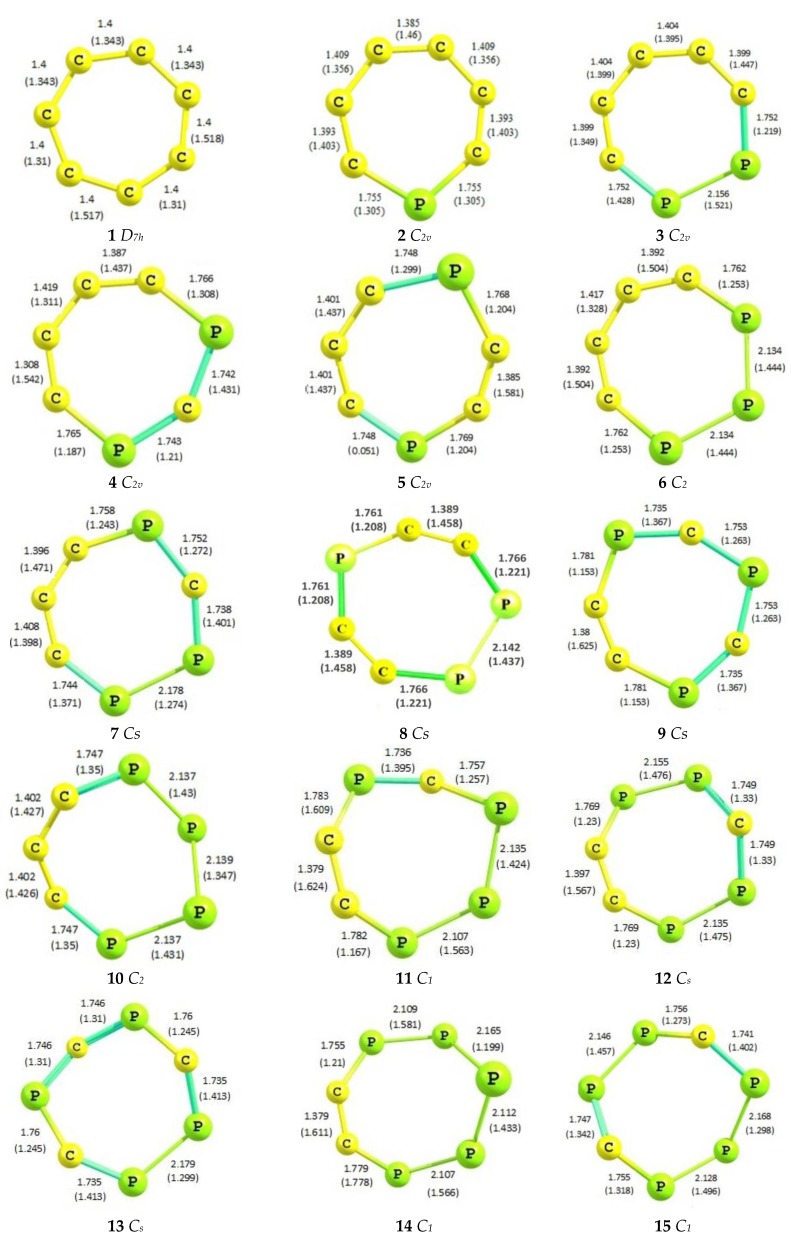
Optimized geometries of tropylium ion and its mono- and poly-phosha analogues at the B3LYP/6-31+G(d) level with bond distances (in Å) and Wiberg bond indices (in parenthesis). For better visualization, H atoms have been omitted.

The geometries of different systems optimized at the B3LYP/6-31+G(d) level along with bond lengths and Wiberg bond indices are reproduced in Figure 1.

The compounds **1**–**15** exhibit significant structural delocalization with CC, CP and PP bond lengths approaching the average of the respective double and single bond lengths of the prototype systems (Table 1).

It is further noted that the planarity of the ring is not disturbed with one and two CH/P exchange(s) in the tropylium ion. Thus, systems **2**–**5** are planar with *C_2v_* symmetry. It is in accordance with the earlier results, according to which incorporation of two σ^2^, λ^3^-P atoms in the five-membered ring reduces the ring strain and makes it planar even if a σ^3^-P atom is also present in the α-position [42]. Likewise, pentaphosphole having a σ^3^-P atom was found to be planar [43,44]. Experimentally also, the first aromatic 1*H*-1,2,4-triphosphole having a fully planar structure could be prepared [45]. Three or more CH/P exchanges in the tropylium cation, leading to **6**, **10**, **11**, **12**, **13**, **14** and **15** are accompanied by loss of planarity, with the exception of 1,2,4- (**7**) and 1,3,5-triphosphacycloheptatrienyl (**9**) cations, though this deviation is not much, as evident from the dihedral angles D_h_ C_a_ 30°.

**Figure 2:**
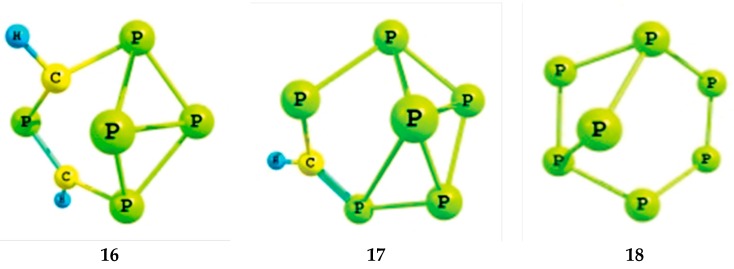
5P (**16**), 6P (**17**) and 7P (**18**) analogues of tropylium ion.

As mentioned earlier, on making five or more CH/P exchanges in the tropylium ion, monocyclic structure is no more stable and it changes into bicyclic (**7P** atoms, **18**) or polycyclic (**5P** atoms, **16**; **6P** atoms, **17**) structures as shown in Figure 2.

### 2.2. Frontier Molecular Orbitals

**Figure 3:**
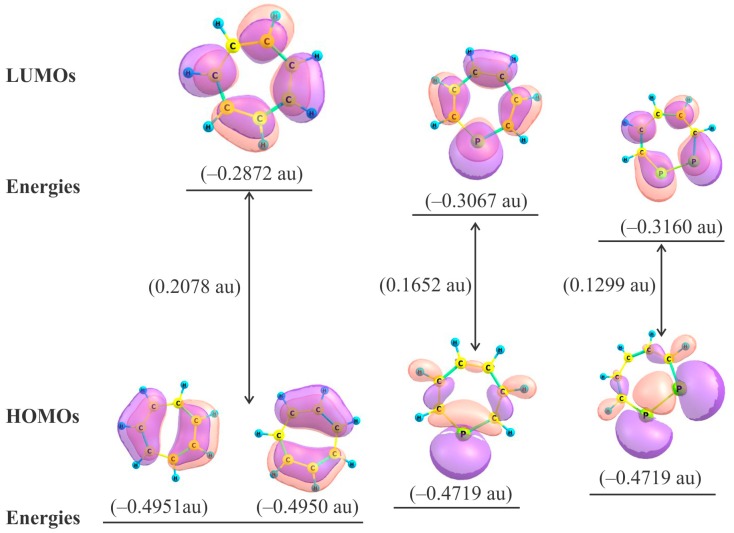
Frontier molecular orbitals (FMOs) of the tropylium cation (**1**); phosphatropylium cation (**2**) and 1,2-diphosphatropylium cation (**3**).

The tropylium cation (**1**) has doubly degenerate HOMOs, whereas, in the case of mono- and poly-phosphatropylium cations, the *p* orbital on the phosphorus atom(s) containing the lone pair constitutes the HOMO. As discussed later, the HOMO − LUMO gap in a molecule can be used as a measure of its aromatic character. In view of this, the frontier molecular orbitals (FMOs) of the tropylium cation (**1**) and two representative phosphatropylium cations (**2** and **3**) along with their energies are shown in Figure 3. It may be noted that, as compared to the tropylium cation, the HOMO − LUMO gap in the phosphatropylium cations is smaller.

**Figure 4:**
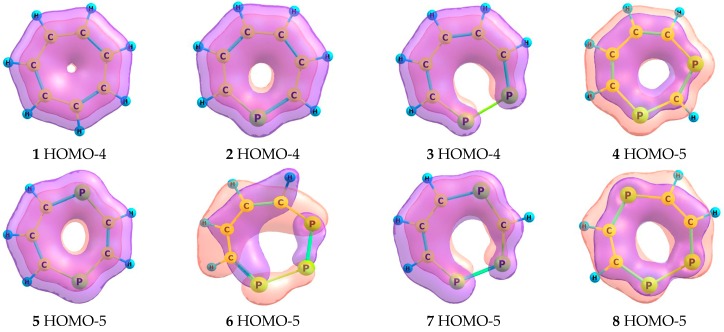

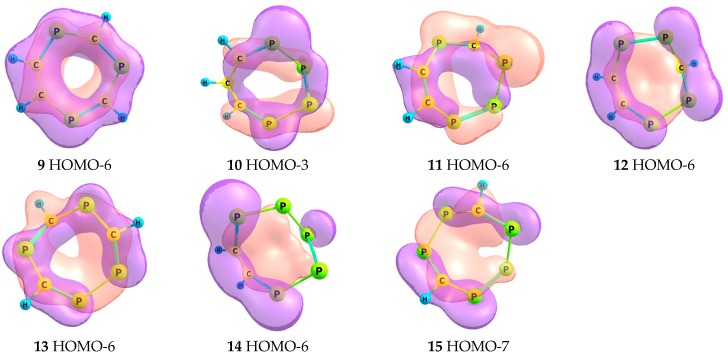
HOMOs of π-symmetry of the tropylium ion and its mono- and poly-phospha analogues at the density functional theory (DFT) (B3LYP/6-31+G(d)) level.

Further FMO analysis reveals that the HOMO-4 orbital of the species **1**, **2**, **3**, HOMO-5 orbital of **4**, **5**, **7**, **8** and HOMO-6 orbital of **9** are the delocalized orbitals of the π symmetry as in their non-phosha analogue i.e., compound **1** (Figure 4). This, in particular, indicates that the aromatic behaviour of these ions is comparable to that of the tropylium ion. However, in the case of the compounds **6**, **10**, **11**, **12**, **13**, **14** and **15**, loss of planarity of the ring reduces the interaction between some of the π orbitals, resulting in diminished delocalization of the π electrons.

### 2.3. HOMO − LUMO Energy Gap

The correlation of the HOMO − LUMO energy gap with kinetic stability and chemical reactivity has been well established [46,47,48,49,50,51,52,53,54]. It can therefore be an important parameter to determine or explain aromaticity in molecules. A large HOMO − LUMO gap means high kinetic stability and low chemical reactivity associated with the aromatic behaviour. It can be well understood on the basis of the fact that it is energetically unfavourable to add electrons to a high-lying LUMO and to extract electrons from a low-lying HOMO to form the activated complex in a potential reaction [46].

This concept was for the first time used to rationalize aromatic character of the polycyclic aromatic hydrocarbons [55]. We extended application of this concept to the present study and have attempted to explain the aromatic behaviour of the tropylium cation and its phospha analogues on the basis of the HOMO − LUMO energy separation and chemical hardness. The chemical hardness has been calculated using Pearson formula (Equation (1)) [56,57]:

(1) η = (εLUMO – εHOMO) / 2

The values of the HOMO − LUMO energy gap (Δε) and chemical hardness (η) are given in Table 2.

A low HOMO − LUMO energy gap (<2.08 × 10^−19^ J) was associated with the antiaromatic character of the dianions of the polycyclic hydrocarbons [58]. From these results, it was extrapolated that the aromatic systems would have a large HOMO - LUMO energy gap. It may be noted that the value of the HOMO − LUMO energy gap for **1** to **15** lies in the range of 3.75 × 10^−19^–9.05 × 10^−19^ J, which indicates aromatic character of these systems. The decrease in HOMO − LUMO energy gap in the case of **6** and **10**–**15** can be attributed to the loss of planarity in these compounds.

**Figure 5:**
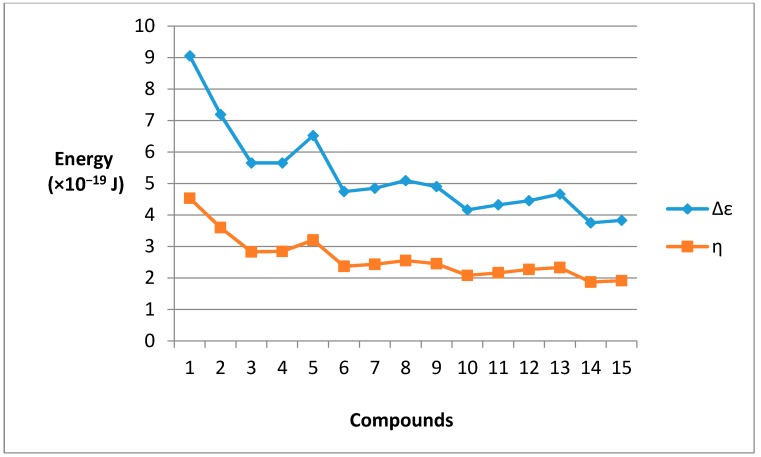
Variation of Δ_HOMO–LUMO_ and chemical hardness in tropylium cation and its mono- and poly-phospha analogues.

It has been shown earlier that an excellent correlation exists between absolute hardness and aromaticity, both increasing almost parallel to each other [56]. In the present case, chemical hardness also varies parallel to the HOMO − LUMO energy gap vis-à-vis aromaticity as shown in Figure 5.

### 2.4. ASE

**Scheme 2:**
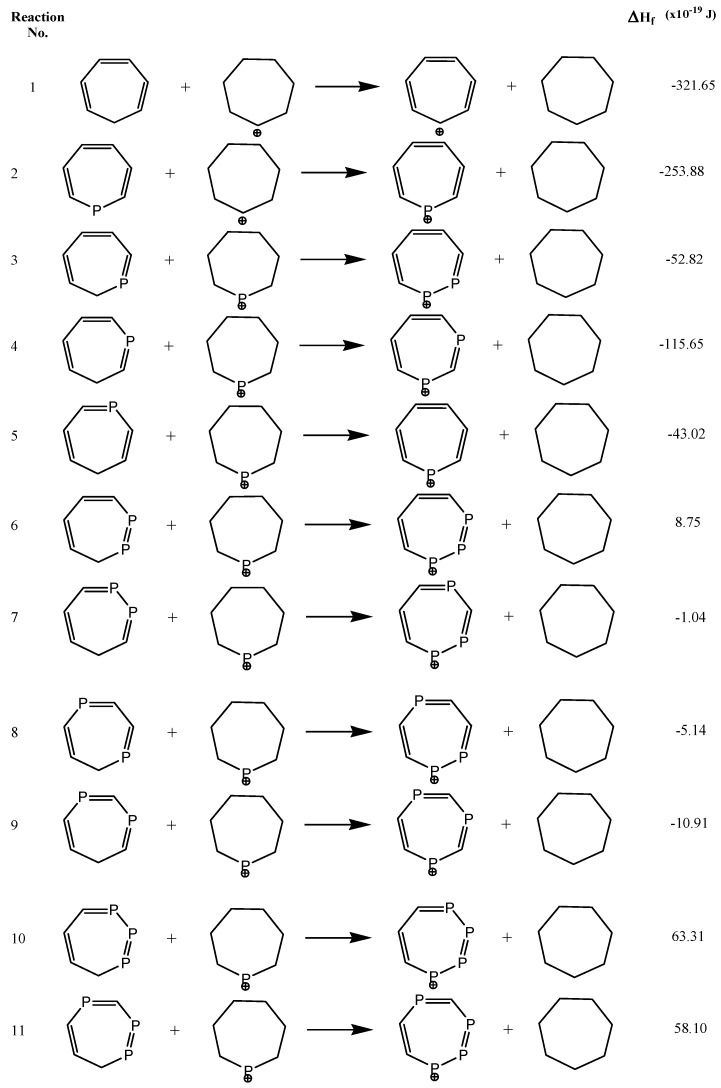
Homodesmotic reactions for aromatic stabilization energy (ASE) determination.

The ASEs of different systems were computed using the following homodesmotic reactions (Scheme 2).

The ASEs of the tropylium ion (**1**) and phosphatropylium ion (**2**) are in conformity with their aromatic character. However, on further CH/P exchange(s), the systems lose planarity as a result of which, ASEs do not change consistently. It is obvious that for the phosphatropylium ions having two or more phosphorus atoms, ASE is no more a reliable indicator of the aromaticity.

### 2.5. Magnetic Criteria

It is well known that a diatropic current is induced in an aromatic system when it is placed in an external magnetic field. This generates a secondary magnetic field causing a deshielding effect at the periphery and a shielding effect inside the ring. Based on this effect, three indicators, namely ^1^H-NMR chemicals shifts [22], NICS [23] and magnetic susceptibility exaltation [59,60] have been used extensively to understand aromaticity. A closely related parameter, magnetic susceptibility anisotropy has also been used to validate aromatic character of different compounds. The aromatic character of the species **1**–**15** is discussed on the basis of all these criteria.

#### 2.5.1. ^1^H-NMR Chemical Shifts

The most common measure of the ring current is the ^1^H-NMR chemical shift, although Schleyer et al. reminded the chemical community of the limitations of proton shifts in the measurement of a ring current [16]. The chemical shift of a proton is the difference of the magnetic shielding of a reference (Tetramethylsilane in the present case) and the proton in question calculated at the same level of theory (Equation (2)):

(2) δ1H =σTMS−σ1H ppm

The ^1^H-NMR chemical shift values of different species calculated are given in Table 3. It is noted that ^1^H-NMR chemical shifts of protons in all of the species **1**–**15**, fall in the aromatic region, confirming their aromatic character. In tropylium ion (**1**), all protons are equivalent and the calculated chemical shift value is δ 9.3. It is noteworthy that on successive CH/P exchanges, the NMR shift values of the protons adjacent to the phosphorus atom(s) appear further downfield, which may be attributed to the stronger electron-acceptor character of the P=C- moiety.

#### 2.5.2. NICS Values

Among the widely used magnetic criteria for aromaticity and anti-aromaticity, NICS proposed by Schleyer and co-workers in 1996 is a simple efficient probe [23]. Subsequently, there have been many refinements and at present NICS(1)_zz_ value is considered to be the most reliable indicator of aromaticity [61]. NICS(0) is defined as the negative value of the absolute shielding computed at the ring centre determined by the average of the heavy atoms coordinates in the ring. NICS(1) is the negative value of the absolute shielding measured 1 Å above the center of the ring, while NICS(1)_zz_ is the out-of-plane component of the absolute shielding estimated in the same position as NICS(1). Rings with highly negative values of NICS are qualified as aromatic by definition, whereas those with positive values are anti-aromatic.

The calculated NICS(0), NICS(1) and NICS(1)_zz_ tensor component values for all species are given in Table 3.

It is noteworthy that these values are negative ranging from −17.54 to −26.42, confirming the aromatic character of the species **1** to **15**.

#### 2.5.3. Magnetic Susceptibility Exaltation

The magnetic susceptibility exaltation (Λ) evaluates the effect of a ring current by comparing the bulk magnetic susceptibility (χ) to the susceptibility of a localized ring system [59,60].

The magnetic susceptibility exaltation with CSGT (continuous set gauge transformations) method values is determined by subtracting the sum of the magnetic susceptibilities of the fragments of cyclic electron delocalization from the values of the respective species (Equation (3)):

(3) $$\Lambda =\chi \text{M}-\chi {\text{M}}^{\prime }$$

The Λ values calculated for different species are given in Table 3. As expected, the exaltation values (Λ) of all these compounds are negative ranging from −20.7 to −54.3 revealing their aromatic character.

#### 2.5.4. Magnetic Anisotropy

Magnetic anisotropy is defined as the difference between out-of-plane and the average in-plane diamagnetic susceptibilities for a ring lying in the (xy) plane [62,63] (Equation (4)):

(4) Δχ=χzz – (1/2)[χxx+χyy]

The values of magnetic anisotropies so calculated are given in Table 3. It may be noted that these values are negative confirming aromatic character of the species.

## 3. Computational Method and Models

All calculations were carried out using a Gaussian 03 suite of programmes [64]. A diffuse function was added to the heavy atoms and geometry optimization of all the systems was done in the gas phase at the B3LYP/6-31+G(d) level of theory. Frequency calculations were done at the same level to determine zero-point energies and to characterize the energy minima.

^1^H-NMR chemical shifts were calculated at the GIAO-B3LYP/6-311++G(d,p)//B3LYP/6-31+G(d) level. NICS values were calculated at the (3,+1) ring critical point of the electron density topology as defined by Bader [65] at the GIAO-B3LYP/6-311++G(d,p)//B3LYP/6-31+G(d) level. Magnetic susceptibilities were calculated at the csgt-B3LYP/6-31+G(d)//B3LYP/6-31+G(d) level and exaltation values were determined by subtracting the sum of the magnetic susceptibilities of the fragments (as explained in the supporting information) calculated at the same level from the value of the respective species.

## 4. Conclusions

On CH/P- exchange in the tropylium ion, aromaticity is sustained up to four carbon atoms. However, with further exchange(s), the system does not remain monocyclic, and it changes to bicyclic and polycyclic systems. Of the various indicators for the aromaticity, the ones based on the magnetic field effect, namely ^1^H-NMR chemical shifts, NICS(1)_zz_, magnetic susceptibility exaltation and magnetic anisotropies are more consistent and confirm the aromatic character of these species.

## Figures and Tables

**Table 1:** Average bond lengths (in Å) at the B3LYP/6-31+G(d) level.

Species	C-C (1.53 Å)	C=C (1.33 Å)	P-C (1.87 Å)	P=C (1.67 Å)	P-P (2.72 Å)	P=P (2.04 Å)
**1**	1.40	1.40	-	-	-	-
**2**	1.41	1.39	1.75	-	-	-
**3**	1.40	1.40	1.75	1.75	2.15	-
**4**	1.41	1.35	1.75	1.75	-	-
**5**	1.40	1.40	1.75	1.75	-	-
**6**	1.40	1.41	-	1.76	2.13	-
**7**	1.40	1.40	1.75	1.75	2.17	-
**8**	-	1.38	1.76	1.74	-	-
**9**	-	1.40	1.76	-	2.13	2.14
**10**	1.40	1.39	1.75	1.75	-	2.14
**11**	-	1.38	1.76	1.74	2.14	2.11
**12**	-	1.40	1.75	1.75	2.14	2.15
**13**	-	-	1.75	1.74	2.17	-
**14**	-	1.38	1.76	-	2.14	2.11
**15**	-	-	1.75	1.75	2.15	2.13

**Table 2:** Frontier molecular orbital energies and hardness of the tropylium ion and its mono- and poly-phospha analogues.

Species	HOMO (a.u.)	LUMO (a.u.)	LUMO − HOMO (a.u.)	Δε (×10^−19^ J)	η (×10^−19^ J)
**1**	−0.4951	−0.2872	0.2078	9.05	4.53
**2**	−0.4719	−0.3067	0.1652	7.19	3.60
**3**	−0.4459	−0.3160	0.1299	5.65	2.83
**4**	−0.4485	−0.3179	0.1306	5.65	2.84
**5**	−0.4650	−0.3176	0.1474	6.52	3.20
**6**	−0.4376	−0.3287	0.1089	4.74	2.37
**7**	−0.4372	−0.3259	0.1113	4.85	2.43
**8**	−0.4431	−0.3264	0.1167	5.09	2.55
**9**	−0.4384	−0.3258	0.1126	4.90	2.45
**10**	−0.4284	−0.3328	0.0956	4.16	2.08
**11**	−0.4332	−0.3341	0.0991	4.32	2.16
**12**	−0.4357	−0.3335	0.1022	4.45	2.27
**13**	−0.4331	−0.3261	0.1070	4.66	2.33
**14**	−0.4271	−0.3412	0.0859	3.75	1.87
**15**	−0.4272	−0.3394	0.0878	3.83	1.91

**Table 3:** ^1^H-NMR chemical shifts, nucleus-independent chemical shift (NICS), magnetic susceptibility exaltation, magnetic anisotropy and aromatic stabilization energy ASE values of tropylium ion and its phospha analogues.

Species	^1^H-NMR Chem. Shift (δ)		NICS(0)	NICS(1)	NICS(1)_zz_	Ʌ	χ_anis_	ASE (×10^−19^J)
**1**	H1	9.39	−6.30	−9.52	−26.42	−20.72	−79.89	−321.65
	H2	9.34						
	H3	9.38						
	H4	9.38						
	H5	9.35						
	H6	9.34						
	H7	9.36						
**2**	H2	11.14	−5.28	−8.72	−23.78	−34.05	−102.65	−253.88
	H3	9.39						
	H4	9.24						
	H5	9.24						
	H7	11.14						
	H6	9.39						
**3**	H3	11.53	−4.32	−7.78	−21.73	−32.72	−116.79	−52.82
	H4	9.31						
	H5	9.37						
	H6	9.31						
	H7	11.53						
**4**	H2	13.16	−4.19	−7.78	−20.70	−35.95	−105.56	−115.65
	H4	10.86						
	H5	9.18						
	H7	10.86						
	H6	9.15						
**5**	H2	11.11	−4.34	−7.92	−21.31	−36.66	−109.68	−43.02
	H3	11.11						
	H5	11.18						
	H6	9.24						
	H7	11.18						
**6**	H4	10.98	−4.69	−8.00	−21.26	−39.93	−121.50	8.75
	H5	9.36						
	H6	9.36						
	H7	10.98						
**7**	H3	13.59	−3.26	−6.97	−18.62	−33.49	−119.21	−1.04
	H5	11.19						
	H6	9.09						
	H7	11.56						
**8**	H3	11.27	−3.59	−7.31	−19.91	−35.64	−121.98	−5.14
	H4	11.18						
	H6	11.18						
	H7	11.27						
**9**	H2	13.02	−2.99	−6.75	−17.54	−37.45	−107.94	−10.91
	H4	13.02						
	H6	10.78						
	H7	10.78						
**10**	H5	10.84	−5.06	−8.06	−20.06	−44.93	−115.3	63.31
	H6	9.12						
	H7	10.84						
**11**	H4	12.73	−4.68	−8.13	−20.46	−45.94	−109.43	58.10
	H6	10.61						
	H7	10.45						
**12**	H3	12.31	−5.18	−8.96	-23.86	−47.65	−114.03	89.09
	H6	11.03						
	H7	11.03						
**13**	H3	13.06	−2.70	−6.99	−17.83	−38.43	−105.24	45.17
	H5	12.74						
	H7	13.06						
**14**	H7	11.06	−7.83	−9.79	−22.83	−54.31	−107.81	93.69
	H6	10.12						
**15**	H3	12.63	−3.25	−7.54	−19.96	−42.88	−105.24	173.95
		H7	11.54						
